# Supplementary material for: Saccharomyces boulardii (CNCM I-745) improves intestinal damage in sepsis by remodeling intestinal flora structure
Source: Front Cell Infect Microbiol. 2025 Jul 31;15:1602792. doi: 10.3389/fcimb.2025.1602792 (PMC12350329; doi:10.3389/fcimb.2025.1602792)
Supplement: Supplementary file 1 [file DataSheet1.zip › summary/summary/8_Advanced_analyse/2_PICRUSt2_result/KEGG/Group.AW4_vs_AW0/PICRUSt2_KEGG_level2_Group.AW4_vs_AW0_diff.pdf]

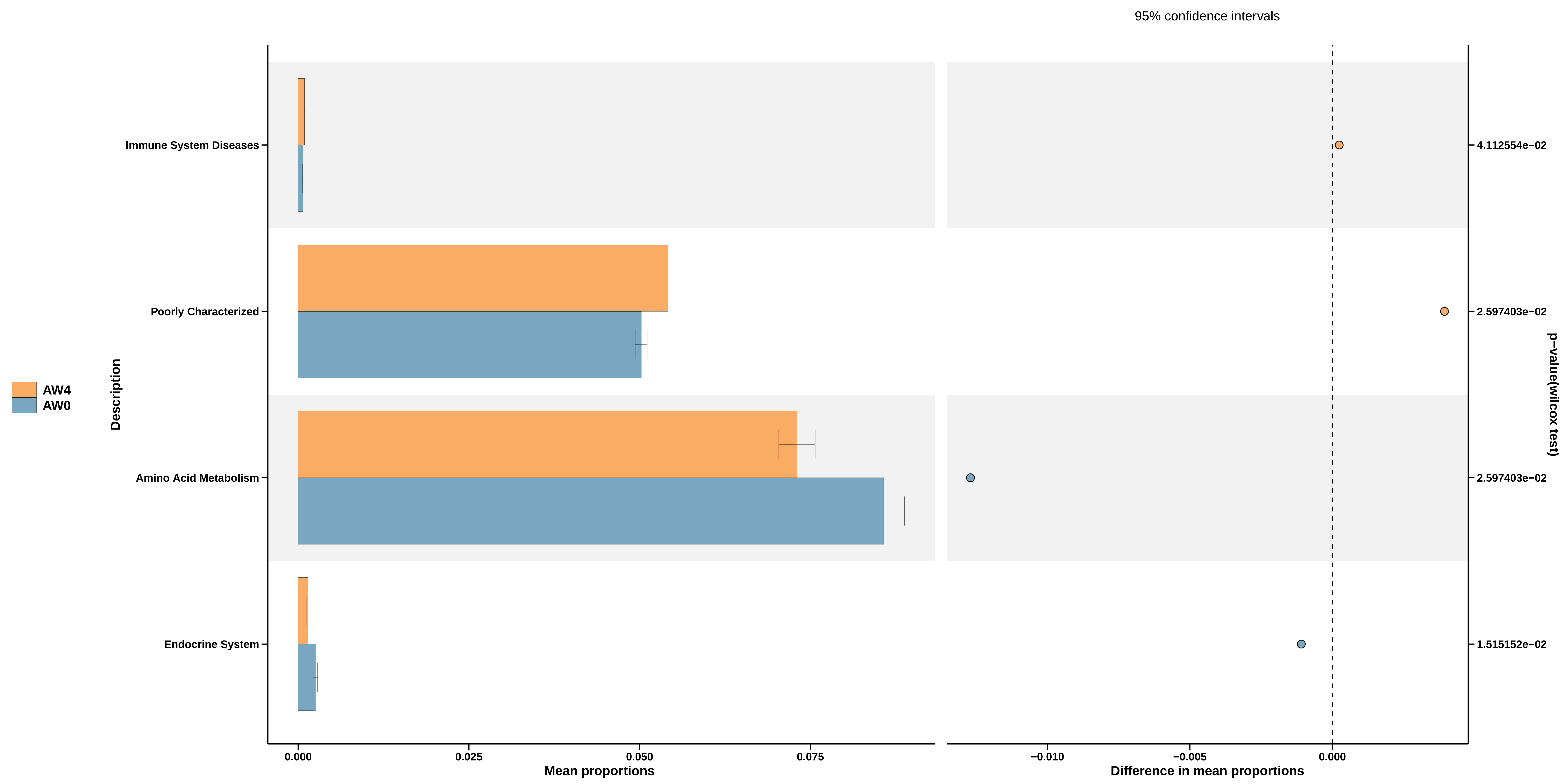

95% confidence intervals
Immune System Diseases
Poorly Characterized
Amino Acid Metabolism
Endocrine System
Description
AW4
AW0
0.000
0.025
0.050
0.075
Mean proportions
−0.010
−0.005
0.000
Difference in mean proportions
4.112554e−02
2.597403e−02
2.597403e−02
1.515152e−02
p−value(wilcox test)
